# Simulating human well-being with large language models: Systematic validation and misestimation across 64,000 individuals from 64 countries

**DOI:** 10.1073/pnas.2519394122

**Published:** 2025-11-26

**Authors:** Pat Pataranutaporn, Nattavudh Powdthavee, Chayapatr Archiwaranguprok, Pattie Maes

**Affiliations:** ^a^Media Lab, Massachusetts Institute of Technology, Cambridge, MA 02139-4307; ^b^Division of Economics, Nanyang Technological University, Singapore 639818, Singapore

**Keywords:** subjective well-being, large language models, global inequality, artificial intelligence, life satisfaction

## Abstract

Subjective well-being is central to economic, medical, and policy decision-making. We evaluate whether large language models (LLMs) can provide valid predictions of well-being across global populations. Using natural-language profiles from 64,000 individuals in 64 countries, we benchmark four leading LLMs against self-reports and statistical models. Unlike regressions, which estimate relationships from survey data, LLMs draw only on individual characteristics (e.g., sociodemographic, attitudinal, and psychological factors) together with associations encoded during pretraining, rather than from the survey’s subjective well-being responses. They produced plausible patterns consistent with known correlates such as income and health, but systematically underperformed relative to regressions and showed the largest errors in underrepresented countries, reflecting biases rooted in global digital and economic inequality. A preregistered experiment revealed that LLMs rely on surface-level linguistic associations rather than conceptual understanding, leading to predictable distortions in unfamiliar contexts. Injecting contextual information partly reduced—but did not remove—these biases. These findings demonstrate that while LLMs can simulate broad correlates of life satisfaction, they fail to capture its experiential and cultural depth. Accordingly, they should not be used as substitutes for human self-reports of well-being; doing so would risk reinforcing inequality and undermining human agency.

## Significance

Governments and international organizations increasingly view subjective well-being as a core measure of progress, yet high-quality data remain costly, uneven, and infrequent. Large language models (LLMs) have been proposed as scalable supplements that could generate population-level predictions from existing demographic or administrative information. Using data from 64 countries, we show that although LLMs reproduce some broad correlates of life satisfaction, they systematically underperform relative to traditional models and make the largest errors in underrepresented settings. Controlled experiments reveal that they rely on linguistic associations rather than conceptual understanding, producing systematic biases that mirror global inequalities. These results demonstrate that LLMs are not valid or ethical substitutes for direct human self-reports and should only be used as diagnostic, rigorously validated research tools.

Subjective well-being, particularly overall life satisfaction, is increasingly recognized as a core measure of human flourishing and a critical input into economic, medical, and policy decision-making (1–4). While many countries now collect well-being data (5–7), coverage remains uneven, especially in lower-resource settings where high-quality, nationally representative surveys are costly, logistically difficult, or unavailable (8). In some contexts, adoption of such surveys may be highly constrained by political resistance or ideological skepticism about the value or legitimacy of subjective indicators. This leaves billions of people—often those most vulnerable to policy decisions—effectively invisible to well-being monitoring systems. Even in data-rich contexts, surveys are infrequent and vulnerable to respondent fatigue (9) and social desirability bias (10), limiting their ability to capture dynamic changes or provide timely insights during crises when rapid policy responses are most needed. These limitations have motivated interest in complementary approaches for tracking well-being accurately, ethically, and at scale.

Large language models (LLMs) have been proposed as one such possibility, aiming to complement survey data and established inferential methods such as psychometric scaling and imputation by drawing on routinely collected demographic or administrative information. Whether such approaches can capture subjective well-being with sufficient accuracy, and without introducing systematic bias, is the question we examine here. Recent work shows that LLMs can generate plausible associations in other domains—such as identifying medical risk factors from clinical records (11, 12) and forecasting political preferences from demographic and attitudinal profiles—but these applications involve patterns that are relatively well-defined and objective (13, 14). Predicting subjective well-being, however, is a more demanding challenge. Life satisfaction is multidimensional and context-dependent across individuals and cultures, making it a stringent test of whether LLMs can move beyond linguistic associations to capture complex human experiences that belong inherently to those individuals. Yet LLMs are trained on vast corpora that already contain widely established determinants of well-being—including income, education, health, marriage, and freedom—and can combine this knowledge with individual-level traits and expressed values. In principle, this could allow them to simulate life satisfaction scores, even without exposure to actual self-reports. Testing how close—or how far—those simulations fall from human assessments provides a stringent validity check. Understanding these limitations is essential for interpreting whether model-based estimates could ever complement official metrics such as the OECD Better Life Index, the UN Sustainable Development Goals, or national frameworks like those of the UK Office for National Statistics and New Zealand Treasury’s Living Standards Framework (15–17). Our analysis, therefore, treats well-being prediction as a stress test of current models, not a proposal for policy substitution.

This focus is also urgent because AI is already being applied in well-being contexts. AI-driven mental health chatbots such as Woebot and Wysa—and more recently, LLM-based conversational agents like ChatGPT and Replika—are marketed as scalable, personalized tools for psychological support, tailoring outputs to individual users based on their inputs (18, 19). Similar approaches are also used in workplace sentiment analysis, where AI platforms monitor employee morale as proxies for well-being (20). While LLMs are not yet used to predict well-being at a global scale, their expanding role in mental health, workplace analytics, and sentiment dashboards suggests we are edging closer to such applications. However, if these systems systematically misestimate well-being, particularly for marginalized populations, they risk reinforcing inequality and diverting resources away from those who need them most. These developments highlight the need to evaluate LLM performance rigorously and to distinguish empirical capability from aspirational marketing. Our study, therefore, treats life-satisfaction prediction as a diagnostic stress test of current models: We examine whether the broad knowledge embedded in LLMs—spanning scientific evidence, policy discourse, and cultural narratives about well-being—allows them to approximate self-reports, and where these approximations fail. We explicitly do not propose LLMs as replacements for surveys. Instead, we show that their systematic errors expose the limits of automated reasoning about human experience, underscoring the need for ethical caution and validation before any practical use.

**Fig. 1.**
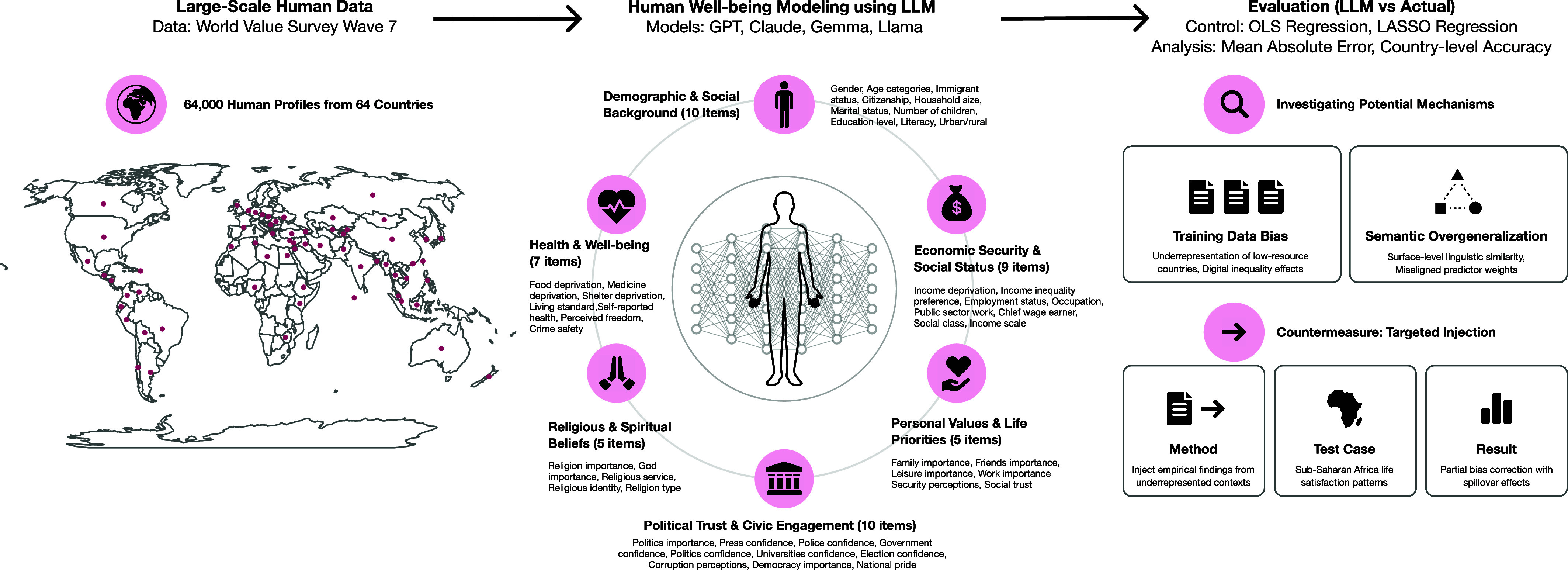
The experimental framework evaluates LLMs’ ability to predict individual life satisfaction across 64 countries, benchmarking LLMs’ performance against traditional statistical approaches using WVS data from 64,000 respondents. The study also examines semantic overgeneralization mechanisms and tests targeted injection methods to determine whether systematic biases in underrepresented regions can be mitigated.

To evaluate these issues, we benchmark four LLMs—GPT, Claude, LLaMA, and Gemma—against ordinary least squares (OLS) and Lasso regressions in predicting life satisfaction across 64 countries using World Values Survey (WVS) data (N = 64,000, with 1,000 randomly selected individuals per country). For each individual, we extracted a comprehensive set of socioeconomic, attitudinal, and psychological variables across six key dimensions: 1) Demographic & Social Background (10 items), 2) Economic Security & Social Status (9 items), 3) Health & Personal Well-being (7 items), 4) Personal Values & Life Priorities 5 items), (5) Religious & Spiritual Beliefs (5 items), and 6) Political Trust & Civic Engagement (10 items) (see *SI Appendix*, Table S1a for the complete list of covariates). Using these dimensions, we created models for each approach. We compare models on mean absolute error (MAE) between predicted and observed life satisfaction, examining both overall accuracy and performance across the life satisfaction scale. We also assess how well LLMs reproduce established correlates of well-being, such as income, education, health, and perceived freedom, and whether they preserve or compress cross-country variation. The illustrative abstract can be found in Fig. 1.

Against this benchmark, our analysis reveals two main sources of error: bias in training data and semantic overgeneralization. Because LLMs are mostly trained on texts from wealthier, digitally well-represented countries, their predictions disproportionately reflect the values and experiences of those contexts, leading to systematic misestimation in low-resource and digitally marginalized regions (21–25). LLMs also depend heavily on linguistic proximity: They overemphasize conceptually prominent but empirically weak predictors (e.g., education, democracy) while underweighting stronger but subtler ones (e.g., income, perceived freedom). This semantic overreach is especially problematic in cross-cultural settings, where linguistic salience may diverge from lived experience.

Our study contributes in three ways. First, we provide systematic, cross-country benchmarking of LLM performance in predicting life satisfaction, showing that they underperform relative to traditional statistical models, especially in disadvantaged contexts. Second, we develop diagnostic tests for the mechanisms underlying these errors, demonstrating how linguistic shortcuts and training imbalances produce structural biases. Third, we clarify the ethical and empirical limits of applying LLMs in human-centered domains: Although they can reveal how model training shapes apparent correlations, they are not appropriate substitutes for direct human self-reports. Together, these findings advance both the science of well-being and the evaluation of how LLMs generalize—and fail to generalize—across diverse human populations.

## Results

### Benchmarking LLM Performance against Statistical Models.

In our preregistered experiments, we evaluated the ability of four LLMs—GPT-4o mini, Claude 3.5 Haiku, LLaMA 3.3 70B, and Gemma 3 27B—to predict individual life satisfaction across 64 countries using observational data from the WVS (N = 64,000; 1,000 respondents per country). For benchmarking, we compared out-of-sample LLM predictions to traditional statistical models, including OLS and Lasso regressions, and to participants’ self-reported life satisfaction scores. Unlike OLS and Lasso, which estimate coefficients from the WVS sample, LLMs were prompted only with respondents’ sociodemographic and attitudinal profiles (e.g., income, education, health, perceived freedom) and asked to provide a predicted score on the 0 to 10 life satisfaction scale. Their predictions thus reflect associations embedded in pretraining corpora rather than relationships fitted within the survey itself.

**Fig. 2.**
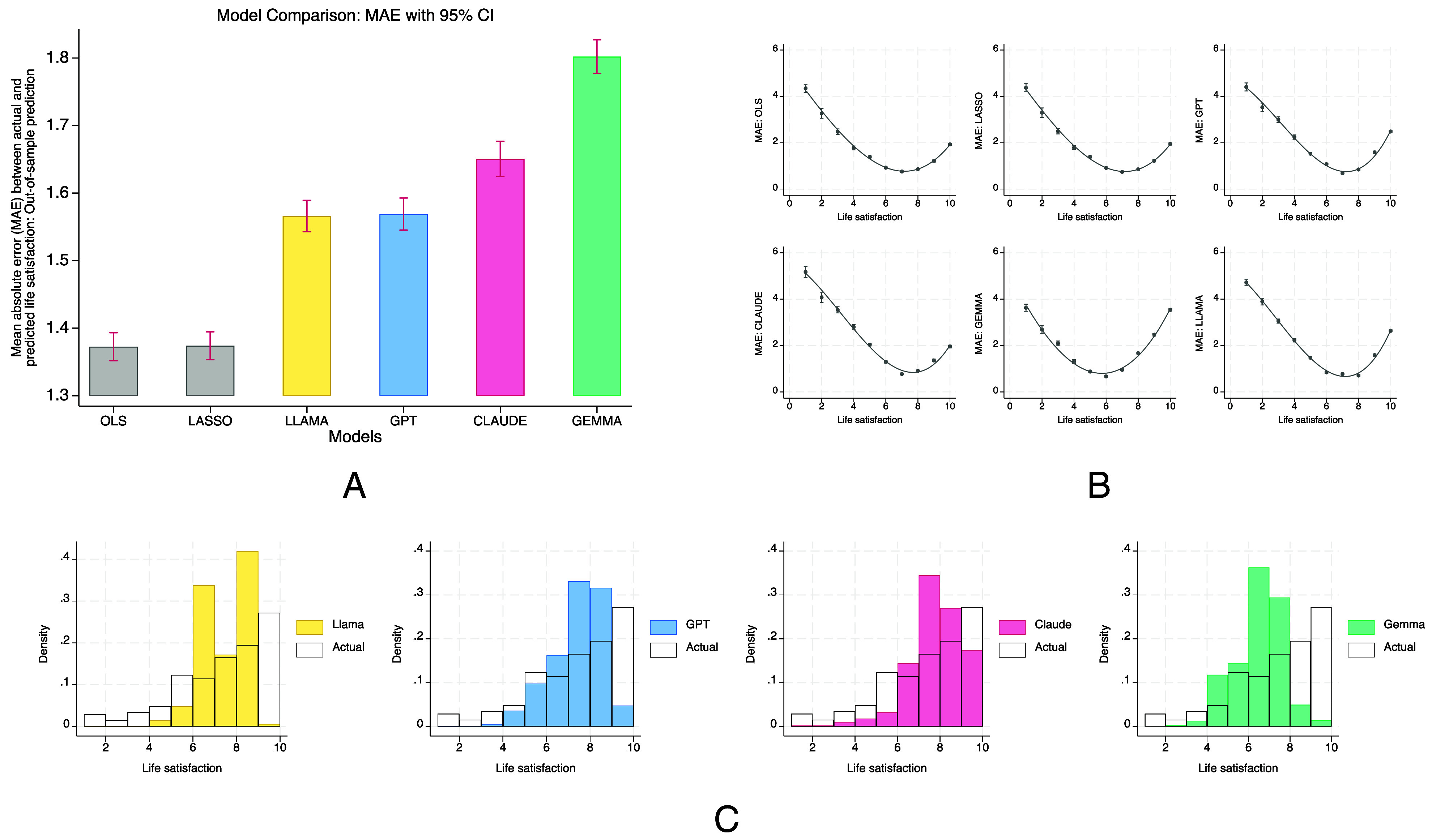
Panel (*A*)—Out-of-sample MAE for OLS and Lasso trained on observed life satisfaction (training set) and for LLM predictions; all values are evaluated on the held-out test set. Error bars show 95% CI. Panel (*B*)—MAE by bins of observed life satisfaction (test set) for regression and LLMs (20% test split). Panel (*C*)—Distributions of i) observed life satisfaction and ii) LLM-predicted life satisfaction in the test set.

Across all models, OLS and Lasso achieved the lowest MAEs, both at 1.37 (95% CI: 1.35 to 1.39). In contrast, all LLMs—LLaMA, GPT, Claude, and Gemma—performed substantially worse, with MAEs ranging from 1.57 (LLaMA and GPT; 95% CI: 1.54 to 1.59) to 1.80 (Gemma; 95% CI: 1.78 to 1.83). These are not minor inaccuracies: an average error difference of 0.43 points, or 31%, relative to OLS, is comparable to the well-documented impact of major life events such as unemployment or marriage (26). Beyond mean accuracy, error rates were unevenly distributed across the life satisfaction scale. As shown in Fig. 2 (Panels *B* and *C*), all models displayed a U-shaped pattern, with the largest errors among individuals at the margins of the distribution (particularly those reporting ≤4), and the lowest errors among those in the mid-to-high range (6 to 8). This pattern was most pronounced for LLMs, suggesting a systematic weakness in predicting life satisfaction among the least satisfied respondents—precisely the groups often of greatest concern to policy.

### Overestimation and Underestimation of Life Satisfaction Predictors.

**Fig. 3.**
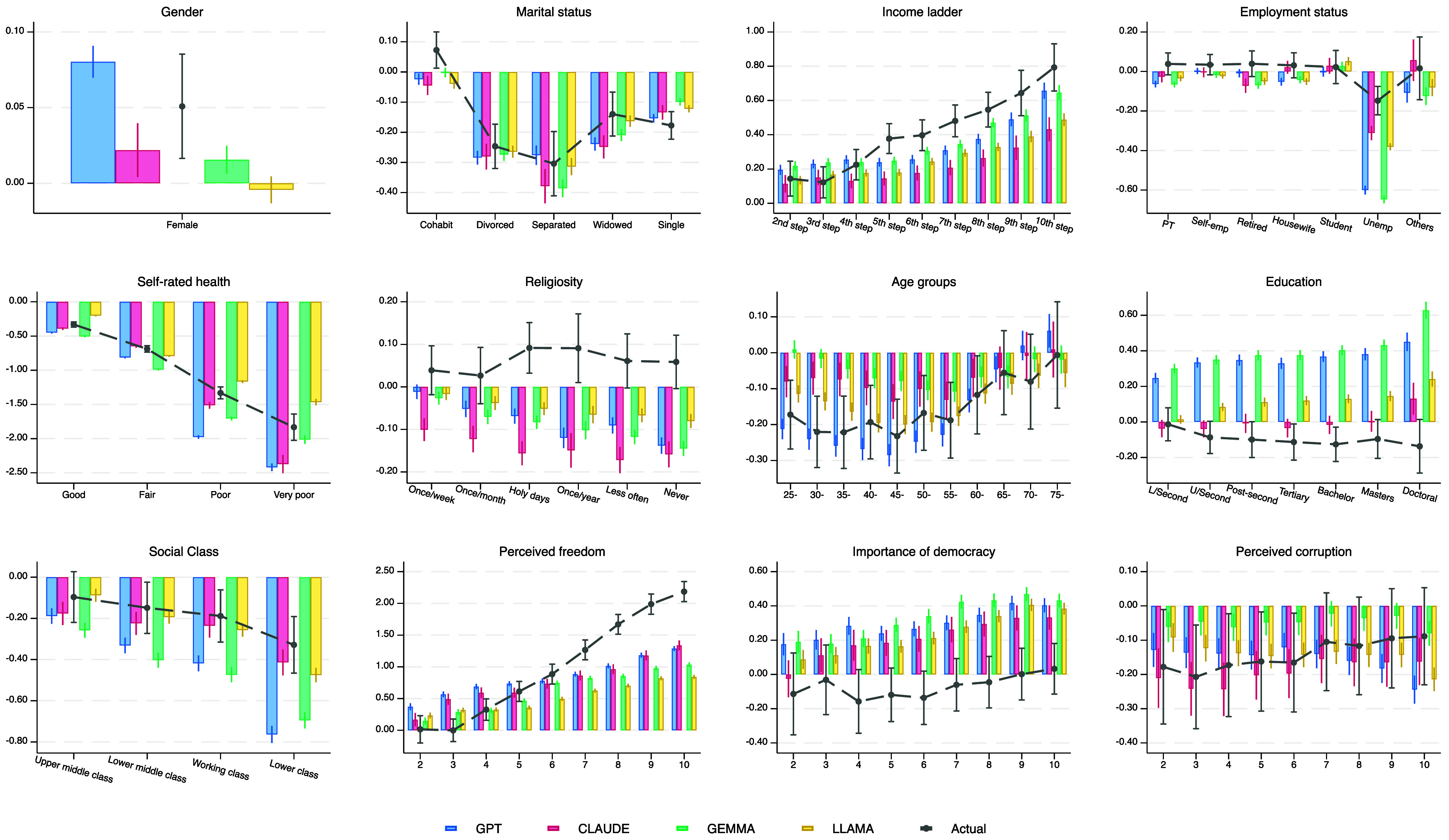
Coefficient plot of socioeconomic and attitudinal predictors of observed and LLM-predicted life satisfaction. Reference categories are male (gender), married (marital status), first step of the income ladder (income), full-time employed (employment), very good (self-rated health), more than once a week (religiosity), under 25 (age), no formal education (education), upper class (social class), little perceived freedom (perceived freedom), democracy is not important (importance of democracy), and zero corruption (perceived corruption). Estimates are obtained from OLS models with country fixed effects and robust SE clustered at the country level. 95% CI are displayed.

To probe the sources of misestimation at the extremes, we examined which individual-level predictors LLMs misrepresent. Fig. 3 contrasts OLS coefficients from observed survey data (dotted lines) with those implied by LLM-generated estimates (colored bars).

Although LLMs recovered some expected associations between well-being and its correlates—for instance, predicting lower life satisfaction for those with poor health or unemployment—several important discrepancies were evident. Perceived freedom, a strong positive correlate of life satisfaction in the data, was consistently underweighted, indicating that LLMs fail to represent the psychological costs of low autonomy. Income gradients were also compressed: Whereas survey data show a steep increase from the 2nd to the 10th step of the income ladder, all models flattened this relationship. Conversely, LLMs overstated the role of education, producing a pronounced upward-sloping pattern that diverges from the modest or flat effects documented in the literature (27, 28). Democracy followed a similar trend, with LLMs overestimating its importance compared to self-reports. For unemployment and poor health, LLMs at least reproduced the correct direction—both lowering well-being—but exaggerated the magnitude of these effects. Overall, these discrepancies show that while LLM outputs resemble known relations in sign, they misjudge their relative significance, relying on linguistic associations rather than on empirically grounded patterns. Finally, it is important to note that the plausible associations observed in the LLM regressions reflect knowledge encoded during pretraining, since the models never had access to actual life satisfaction responses in the WVS.

### LLMs Attenuate Country-Specific Variation.

**Fig. 4.**
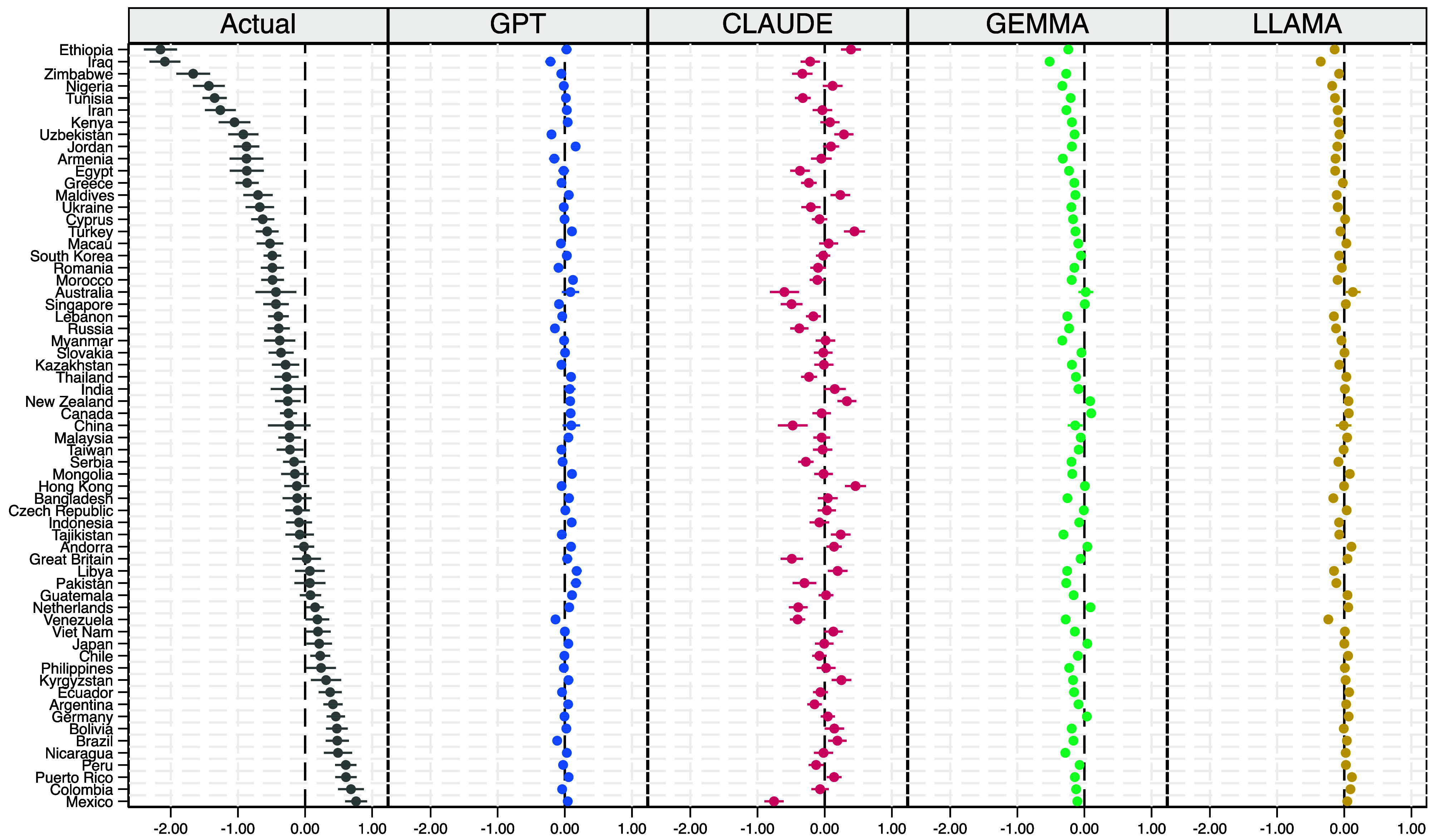
Coefficient plot of country fixed effects from regressions of observed and LLM-predicted life satisfaction (as in Fig. 3). Estimates are relative to the United States, the reference category. 95% CI are shown.

Having examined individual-level predictors, we next assessed whether LLMs capture systematic country-level differences in life satisfaction that remain unexplained after accounting for observed factors such as income, health, and employment—potentially reflecting cultural, linguistic, or institutional variation. Fig. 4 presents the estimated country fixed effects for both actual and LLM-predicted outcomes, adjusted for individual-level characteristics such as income, health, education, and employment. In the observed data, country effects varied widely—from –2.1 to +1.0—highlighting substantial cross-national heterogeneity beyond compositional factors. Fixed effects are estimated relative to the United States, which serves as the reference category. Because each country contributes equally to the dataset (1,000 respondents; N = 64,000), the choice of baseline is purely conventional and does not affect substantive interpretation. Nations such as Mexico, Colombia, and Peru reported significantly higher average life satisfaction than the United States (reference group) and most other countries, consistent with prior findings from Latin America (29). Conversely, countries like Ethiopia, Iraq, and Zimbabwe recorded the lowest adjusted scores.

All four LLMs substantially understated this variation. Fixed effects clustered tightly around zero, typically between –0.5 and +0.5, with LLaMA producing the narrowest range (±0.2). Extreme cases were notably misrepresented: Mexico, Colombia, and Peru were assigned near-zero or even negative effects, while Ethiopia, Iraq, and Zimbabwe appeared only modestly below average. Claude and Gemma produced slightly more dispersion than GPT or LLaMA, but none reproduced the magnitude or structure of cross-country differences observed in the actual data. This attenuation has important implications: If applied to global monitoring, such flattening would risk obscuring meaningful cultural or regional differences—such as the well-documented Latin American “happiness advantage”—and could mislead conclusions about inequality in well-being. Although it is theoretically possible that the compression reflects measurement noise in self-reports, the persistence of these patterns across repeated surveys and prior studies indicates that the observed heterogeneity is genuine. Moreover, the fact that attenuation persists even when the country of residence is explicitly included implies a deeper limitation: Current LLMs fail to integrate contextual information in ways that capture lived cultural variation. Further robustness checks (*SI Appendix*, Extended Data Fig. S2a) reinforce this point, showing substantial cross-national disparities in prediction errors, ranging from the Netherlands to Ethiopia for GPT and LLaMA, Malaysia to Ethiopia for Claude, and Iraq to Mexico for Gemma.

Taken together, these findings show that LLMs misestimate the drivers of life satisfaction, overstating the effects of variables like poor health, unemployment, and education while compressing gradients for income and perceived freedom. They also fail to reproduce the wide cross-national variation observed in survey data, despite being given country information. These distortions indicate that current models recover only broad associations and miss the heterogeneity central to understanding individual well-being, suggesting they rely on semantic shortcuts rather than empirically grounded patterns—a mechanism we examine next.

### Training Data Bias and Semantic Generalization as Sources of Misestimation.

Why do LLMs recover broad correlates of well-being yet misestimate their relative importance and compress cross-national variation? We consider two interrelated mechanisms: training data bias and semantic overgeneralization.

#### Training data bias.

**Fig. 5.**
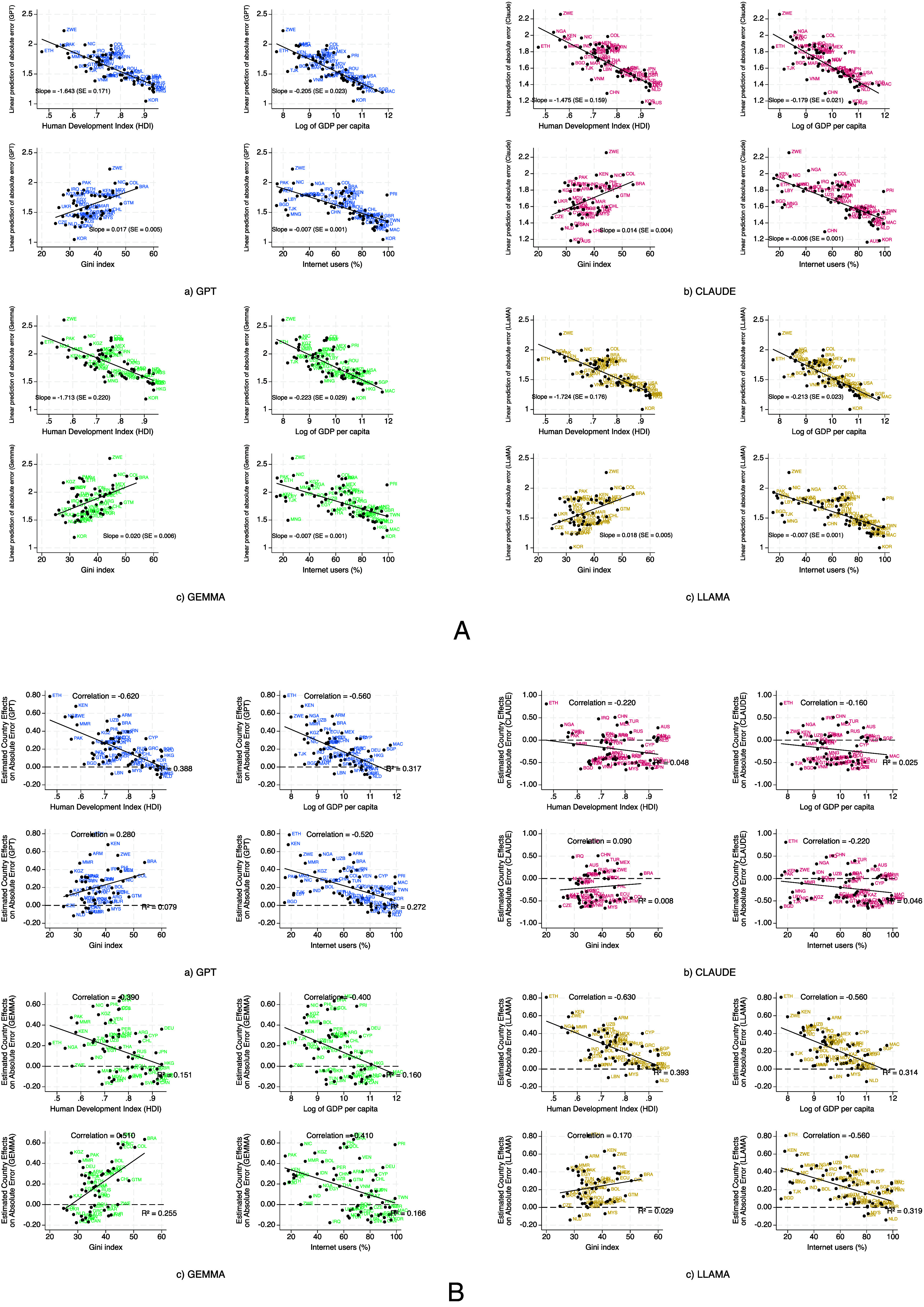
The LLMs reported in the plot include (*A*) GPT, (*B*) Claude, (*C*) Gemma, and (*D*) Llama. Panel (*A*)—Two-way scatter plots showing country-level averages of fitted values from regressions of absolute prediction error on individual-level predictors, plotted against macroeconomic indicators. Panel (*B*)—Two-way scatter plots showing estimated country fixed effects from OLS regressions of absolute prediction error on individual-level predictors, plotted against macroeconomic indicators. The macroeconomic indicators include HDI, log GDP per capita, Gini index (income inequality), and internet usage (% of population). Higher values indicate greater systematic error explainable by observed predictors.

Because LLMs are trained on internet-scale corpora dominated by high-income, English-speaking, and digitally connected contexts (30), their predictions tend to reflect the priorities and linguistic patterns of those settings. This leads to systematic underperformance in low-resource or digitally marginalized countries, particularly in how individual-level predictors relate to life satisfaction. To test this, we modeled absolute prediction error as a function of standard variables (income, employment, health) and averaged the fitted values by country. As shown in Fig. 5*A*, errors were smaller in countries with higher HDI, GDP per capita, and internet access, and larger in countries with greater inequality (higher Gini index). In other words, LLMs more closely approximated the structure of predictors in affluent, digitally connected settings than in poorer or unequal ones, reflecting representation bias inherent in global training data.

Beyond these predictor-level misalignments, we also examined whether LLMs reproduce systematic country-specific offsets in life satisfaction that remain after controlling for individual characteristics. Specifically, we regressed the country fixed effects from Fig. 4—which reflect cultural, linguistic, or institutional variation unexplained by personal characteristics—on macrolevel indicators. As shown in Fig. 5*B*, the country fixed effects estimated from GPT, Gemma, and LLaMA predictions were systematically associated with national development indicators: They became increasingly negative in countries with lower HDI, GDP per capita, and internet access. The Gini index showed a weaker but positive association, indicating greater modeling difficulty in more unequal societies. Claude’s fixed effects, by contrast, were largely uncorrelated with these indicators, indicating a flatter, less context-sensitive error profile.

To further examine these biases, *SI Appendix*, Fig. S3a investigates income–life satisfaction gradients across different development contexts. In low-HDI countries, the observed relationship is significantly steeper than in high-HDI countries, indicating a higher marginal value of income. However, all four LLMs predicted nearly identical gradients for both groups, underestimating the effect in low-HDI settings and overestimating it in high-HDI ones. This pattern illustrates that LLMs tend to impose a uniform income–well-being relationship, consistent with training data that overrepresent wealthier contexts, thus systematically overlooking important differences across contexts.

Taken together, these results show that LLMs’ predictive accuracy declines most in underrepresented or digitally marginalized countries, precisely those least visible in internet-scale training data. This bias accounts for both their misestimation of individual-level predictors and the muted country fixed effects observed in these settings, even as their performance remains stronger in affluent, digitally well-represented contexts.

#### Semantic generalization.

Another mechanism behind LLM prediction errors is their tendency to rely on linguistic associations rather than empirically grounded reasoning, leading to extrapolations that diverge from observed relationships. For example, public discourse often frames education as a marker of success, leading models to overstate its effect—even though survey data show weak associations once income, health, and marital status are controlled for (28). Similarly, the psychological costs of unemployment, prominent in Western narratives, appear overgeneralized. These cases suggest that while LLMs often capture the direction of key predictors, they miscalibrate their relative importance by following patterns of semantic salience. More broadly, semantic proximity does not always align with conceptual equivalence: Just as “happiness” and “life satisfaction” overlap linguistically yet differ in psychological structure (31), LLMs may treat distinct predictors or contexts as interchangeable if they co-occur frequently in text. This reliance on surface-level associations raises concerns about their validity in novel or policy-relevant contexts where precise distinctions matter.

**Fig. 6.**
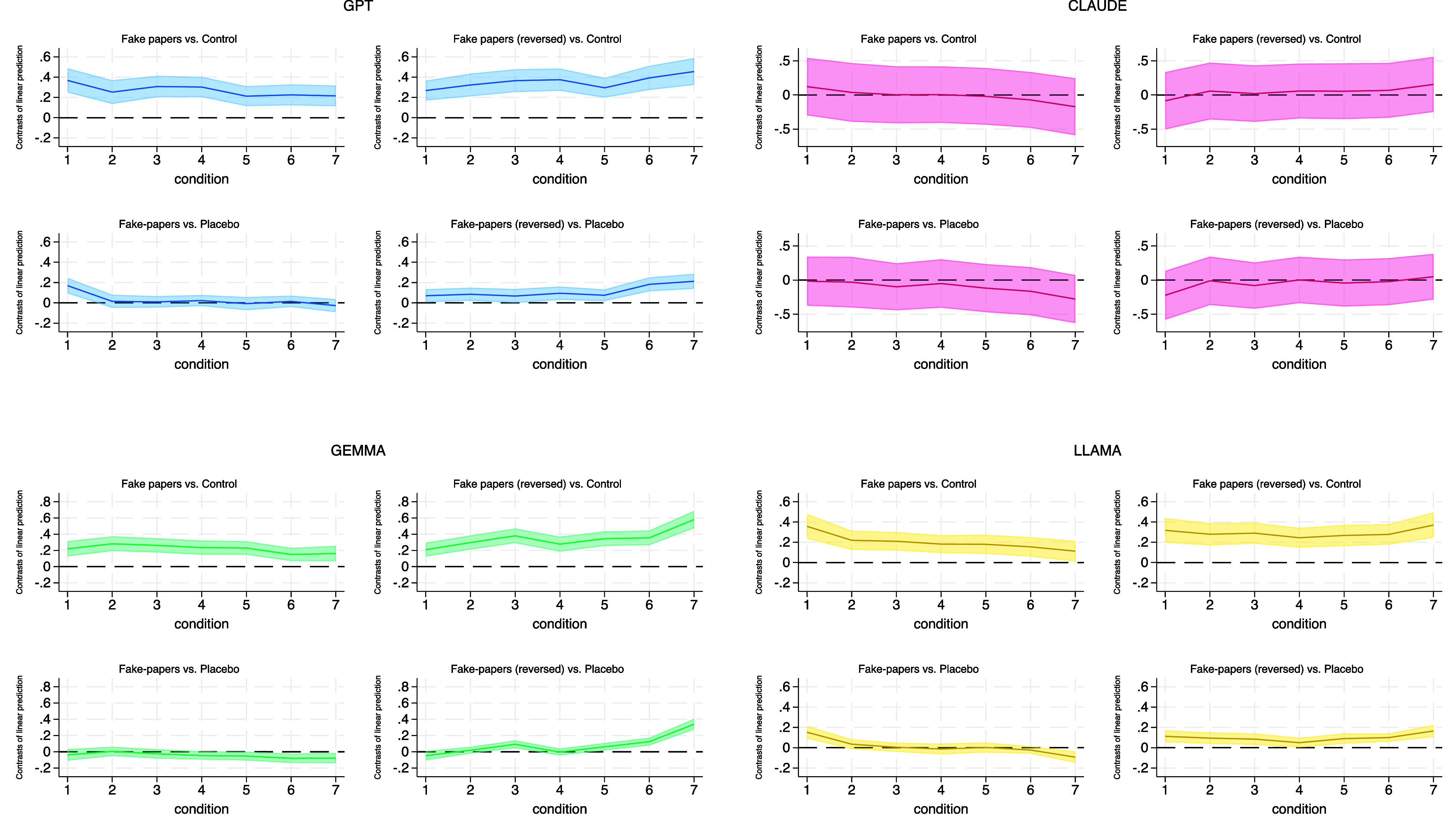
Treatment effects on predicted life satisfaction across semantic conditions and LLMs. Each panel shows estimated differences in predicted life satisfaction between treated and comparison models across seven semantic levels. *Top* row: models with fabricated research papers injection vs. the baseline model (*Left*: original framing; *Right*: reversed). *Bottom* row: same models vs. a placebo model with an injection of unrelated synthetic content. Only conditions 1 (positive) and 7 (negative) were seen during context injection; conditions 2 to 6 represent unseen, semantically interpolated prompts. Shaded areas show 95% CI. All regressions control for intervention fixed effects. Systematic deviations across intermediate conditions indicate semantic generalization beyond the trained endpoints.

To test whether LLMs rely on semantic proximity when generalizing, we designed a randomized experiment with synthetic interventions. Each intervention introduced a pair of fictional variables, one positively and one negatively associated with life satisfaction (e.g., “listening to unicorn voices” vs. “having a dinosaur companion”). These endpoints were linked by five intermediate prompts forming a continuous semantic gradient. Only the endpoints were provided to the models; the intermediates were novel, allowing us to test whether predictions interpolated purely along semantic lines. We present our findings in Fig. 6.

Across 2,800 prompts per model, GPT, Gemma, and LLaMA displayed clear semantic interpolation: Predicted life satisfaction varied smoothly across the gradient, despite the intermediates never being shown. Claude also shifted in the direction implied by the injected information, but its gradient was flatter and statistically indistinguishable from control conditions. Reversing the mapping inverted the gradient in all models, confirming that predictions tracked injected semantics rather than inherent features of the text. Placebo controls using irrelevant but stylistically similar phrases showed no gradient, ruling out generic exposure effects.

The strength of these interpolation patterns varied: GPT and Gemma showed sharp, consistent gradients, LLaMA’s responses were shallower and noisier, and Claude generalized only weakly. Taken together, these findings provide direct evidence that LLMs extrapolate along semantic associations rather than abstract reasoning. While this enables them to capture broad linguistic patterns, it also renders them prone to semantic overreach—a mechanism consistent with their overestimation of education and democracy and their underestimation of subtler correlates such as income and perceived freedom.

In summary, these experiments show that LLMs often treat linguistic similarity as if it implied conceptual equivalence. This strategy can be efficient in familiar domains but introduces risks in novel or policy-sensitive settings where linguistic cues diverge from empirical reality. Models that generalize more aggressively—like GPT and Gemma—are highly responsive to injected signals but also more prone to semantic overreach. By contrast, more conservative models—like Claude—maintain tighter semantic boundaries but at the cost of weaker responsiveness. These dynamics reveal a trade-off between the breadth of generalization and the preservation of conceptual fidelity. They also suggest that the success of injection varies across models and contexts, depending on how salient or conceptually anchored the injected information is. Building on this, we next use Claude as a test case to examine whether selective injection can correct specific mispredictions while minimizing unintended spillover effects.

### Targeted Injection: Testing whether Bias Can Be Corrected.

One of the most noticeable errors in our results was the consistent overestimation of life satisfaction in sub-Saharan countries—especially Ethiopia, Zimbabwe, Nigeria, and Kenya—compared to self-reports. To assess whether this bias could be reduced, we carried out a targeted intervention by introducing a general empirical prompt to the model: that “average life satisfaction in sub-Saharan countries is usually lower than in most other regions.” Building on our earlier finding that Claude generalizes more conservatively than other models, we used it as a test case to limit unintended spillover effects. We then examined how this addition affected predictions across 14 countries (N = 14,000; 1,000 randomly selected participants per country), focusing on both the specific sub-Saharan countries and regions not directly referenced. These included five Latin American countries (Argentina, Brazil, Chile, Colombia, Mexico), where LLMs tend to underpredict life satisfaction, and five others (Canada, Germany, Japan, Myanmar, United States) that show relatively average levels. This approach allowed us to evaluate not only whether the addition corrected the intended misprediction but also whether it caused distortions elsewhere.

**Fig. 7.**
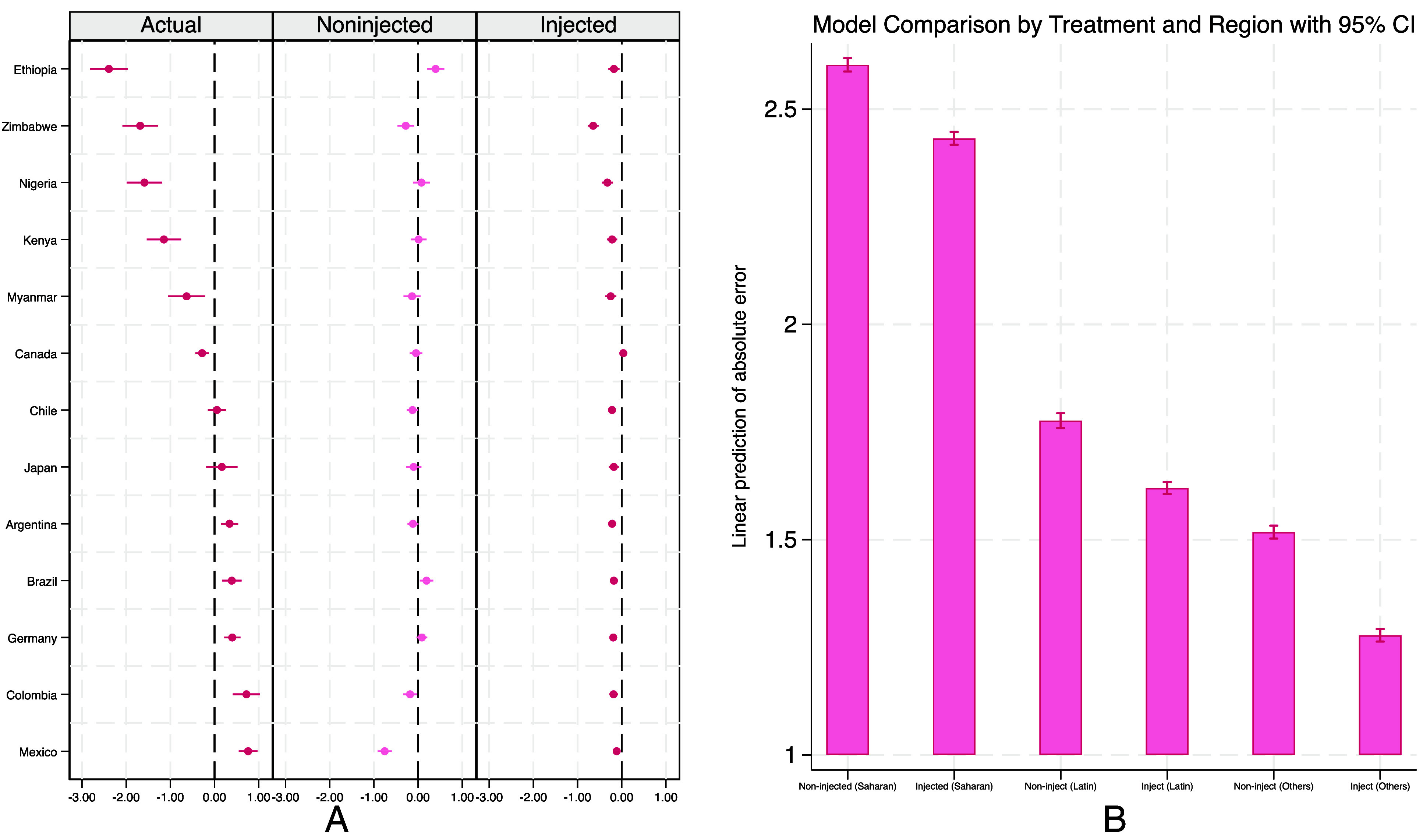
Panel *A*—Coefficient plot of country fixed effects on actual life satisfaction, noninjected LLM predicted life satisfaction, and injected LLM predicted life satisfaction. Reference group: USA. Panel *B*—Linear prediction of absolute error between actual life satisfaction and LLM predicted life satisfaction by treatment (noninjection vs. injection) and region. 95% CI are displayed.

We highlight two key results, presented in Fig. 7. Panel *A* compares country fixed effects on actual self-reported life satisfaction with Claude’s predictions under injected and noninjected conditions. As in earlier analyses, fixed effects represent systematic country-level differences unexplained by individual characteristics. Without injection, Claude predicted life satisfaction in sub-Saharan countries to be comparable to, or even higher than, that of the United States—despite survey data showing considerably lower averages. After injection, all four sub-Saharan countries shifted to significantly lower levels than the reference, moving closer to observed patterns on average. This indicates that the injection effectively shifted predictions in the intended direction, though the gap relative to observed data persisted, signaling only partial correction of the bias.

Panel B examines absolute prediction error by region and treatment condition. Baseline errors were highest in sub-Saharan Africa, where injection produced a substantial reduction. In Latin America and other countries not directly referenced in the injected content, errors were smaller but nevertheless changed following injection. This suggests that even targeted input can influence predictions more broadly, producing both intended and unintended effects.

Taken together, these results suggest that selective injection can improve calibration in underrepresented contexts but also alters the model’s internal weighting of individual predictors, producing spillovers beyond the targeted region. While this highlights the potential of injection as a diagnostic tool, it also underscores its limitations: Factual prompts can modify model outputs, but rarely in a contained way. Careful design, repeated testing, and model-specific evaluation are therefore essential before any such approach could be deployed in practice. For full breakdowns of individual predictors across treatment and region, see *SI Appendix*, Figs. S5a and S7a.

These findings complete our empirical assessment, showing that while targeted interventions can partially reduce bias, they alter model outputs in broader and sometimes unintended ways—a pattern we return to in the Discussion when considering the implications for research and policy.

## Discussion

This study examined the effectiveness of LLMs in predicting life satisfaction across national contexts using large-scale, structured survey data. While the models produced plausible estimates and identified several well-known factors influencing well-being based on encoded pretraining knowledge, their accuracy—when benchmarked against actual WVS life satisfaction reports—varied systematically between countries and predictors. Country-specific effects were muted, and key factors—including income, unemployment, education, and perceived freedom—were often misestimated in both magnitude and significance. This contrasts with LLMs’ strong results on cognitive and linguistic benchmarks (32–34), which reward generalization from textual corpora. In contrast, life satisfaction is a subjective, culturally embedded construct that presents fundamentally different challenges. Our findings therefore represent a systematic evaluation of how LLMs perform when applied beyond language or cognition, in a domain where validity depends on understanding experiential and cross-cultural differences. They show that current LLMs cannot serve as substitutes for people’s own self-reports of well-being and that even superficially convincing estimates can mispresent reality in systematic and ethically consequential ways.

We identified two interrelated mechanisms underlying these patterns: training data bias and semantic generalization. Errors were most pronounced in countries with lower internet penetration, HDI, and GDP per capita, reflecting the influence of resources and digital infrastructure on training exposure. At the same time, our semantic gradient experiments showed that GPT and Gemma interpolated smoothly across fabricated but linguistically related prompts, reversing direction when the polarity of the injection was switched and disappearing under placebo conditions. Claude, by contrast, displayed minimal interpolation, consistent with a more conservative generalization profile. Together, these findings suggest that LLMs generalize along linguistic proximity rather than empirically grounded relationships. This reliance on semantic salience helps clarify why current models appear to perform well on cognitive and linguistic benchmarks, where surface-level associations are rewarded, but struggle with culturally embedded constructs such as life satisfaction, where validity depends on capturing context and heterogeneity. As a result, their apparent coherence should not be mistaken for genuine understanding, particularly in domains that concern subjective human experience.

These mechanisms also explain why predictions can appear plausible while deviating systematically from observed data. In high-resource, digitally well-represented settings, model outputs aligned more closely with survey responses. By contrast, in lower-resource countries, predictions compressed steep gradients—particularly for income and perceived freedom—or amplified culturally salient but weaker predictors such as education and democracy. This misalignment raises risks for policy applications. If used without validation, such models could obscure inequality, overstate the importance of some factors, or understate the needs of marginalized populations. At the same time, their partial recovery of broad correlates highlights that model outputs can sometimes mirror known patterns—but without capturing the underlying mechanisms that give those patterns meaning. Understanding this distinction is critical to preventing apparent precision from masking structural bias.

To explore whether bias can be effectively mitigated, we implemented a targeted injection, introducing an empirical fact about lower average life satisfaction in sub-Saharan Africa. This adjustment shifted Claude’s predictions in the intended direction, reducing errors in the targeted region. Yet the intervention also altered outputs for Latin American countries and influenced how the model weighted individual-level predictors, suggesting that injection rarely produces isolated corrections. Instead, even narrow prompts propagate through internal associations, reshaping behavior more broadly. These findings highlight both the potential and the limitations of injection: It can improve calibration in underrepresented contexts, but it also risks destabilizing internal logic if not carefully designed and validated.

More broadly, these dynamics raise concerns about fairness and epistemic reliability. While predictions may appear coherent, they are often grounded in linguistic shortcuts rather than conceptual validity. Models can therefore inherit biases from dominant discourses or overrepresented literature, reinforcing outdated narratives under the guise of precision. Without explicit safeguards, such artifacts could lend spurious credibility to systematically biased outputs, a risk that becomes ethically significant when findings inform social policy. Yet even imperfect outputs can be informative. Used cautiously, LLMs could help generate hypotheses, flag anomalies between survey waves, or provide provisional insights in data-scarce environments. Nevertheless, such exploratory use should remain tightly bounded by transparency and human oversight, ensuring that automated estimates inform but never replace direct self-reports.

## Limitations

This study has several limitations. First, although our analysis draws on data from 64 countries via the WVS, some populations remain underrepresented—including linguistic minorities and individuals without internet access. This underrepresentation likely contributes to the larger prediction errors observed in low-income and digitally disconnected regions, reinforcing our conclusion that LLM performance is closely tied to the inclusiveness of their training data. Second, the models we evaluated were not fine-tuned for the task of well-being prediction. Their performance, while often plausible, lagged behind traditional statistical models in part because they lacked task-specific calibration. Third, while our semantic interpolation experiment revealed how LLMs generalize on the basis of linguistic similarity, it relied on stylized, synthetic prompts. Future research should test whether the same mechanisms operate when models engage with real-world policy inputs. Fourth, our analysis is cross-sectional; a natural extension would be to examine whether LLMs predict changes in well-being over time, which is often more policy-relevant. Fifth, our analysis focused on cross-country comparisons. Future work should test whether similar biases arise within countries; for example, across linguistic minorities, rural–urban divides, or socioeconomic subgroups. Sixth, our findings suggest that LLMs may amplify associations drawn from widely cited but empirically weak literatures, such as the overemphasis on education, raising questions about how epistemic quality is encoded in training corpora. Future research could examine whether fine-tuned or domain-aligned LLMs reduce such distortions, or whether they inherit and even reinforce the same biases.

Finally, it is important to acknowledge that both LLM-based and regression-based approaches are constrained by the limits of survey data itself. Neither method can capture sudden, idiosyncratic life shocks—such as divorce, bereavement, or trauma—that strongly influence individual well-being but are rarely measured in standard questionnaires. For LLMs, this limitation is deeper: Because they learn from historical text and discourse, they cannot anticipate novel or unrecorded experiences, nor detect shifts that lie outside existing patterns of discourse. These limitations make human self-report indispensable, as they reveal changes no model could infer a priori. Recognizing this ceiling on predictive validity highlights the need for sustained, representative human data collection to ground any model-based estimate of life satisfaction. Yet even these data are imperfect: Self-reports are not free from bias or measurement error, and it is conceivable that model-based estimates might occasionally approximate latent aspects of well-being that individuals misreport or reinterpret. Such possibilities warrant empirical exploration, but they do not alter the ethical and epistemic principle that only human self-reports can reveal how people experience their own lives.

Despite these caveats, our study provides a systematic, cross-country benchmark of LLM performance on a culturally embedded construct like life satisfaction. Addressing these limitations will require more representative training data, domain-specific alignment strategies (e.g., targeted prompt design or fine-tuning), and curated epistemic inputs that prioritize replicable, high-quality sources. Establishing such benchmarks for human-centered outcomes is essential not only to avoid indiscriminate expansion of LLM use but also to clarify the boundaries of what these models can and cannot validly infer. Ensuring that their outputs remain interpretable, accountable, and anchored to human data will be critical for any responsible diagnostic or exploratory use in behavioral or policy research.

## Materials and Methods

### Data.

We used individual-level data from Wave 7 of the WVS (2017–2022), which provides nationally representative samples for 64 countries. From each country, we randomly selected 1,000 respondents to construct a balanced and harmonized cross-national dataset (N = 64,000). All analyses were preregistered prior to data analysis (https://aspredicted.org/3xsf-kx78.pdf for the main analysis, https://aspredicted.org/8c6y-2q55.pdf for the semantic similarities experiment, and https://aspredicted.org/n3v6-2rfq.pdf for the targeted injection experiment). All the codes can be found on https://github.com/mitmedialab/wellbeing-LLM/.

### Life Satisfaction Prediction Using LLMs.

We extracted a comprehensive set of socioeconomic, attitudinal, and psychological variables from each respondent across six key dimensions: 1) Demographic & Social Background (10 items), including gender, age categories, immigrant status, citizenship, household size, marital status, number of children, education level, literacy, and urban/rural residence; 2) Economic Security & Social Status (9 items), including income deprivation, living standards, income inequality preferences, employment status, occupation, public sector work, chief wage earner status, social class, and income scale; 3) Health & Personal Well-being (7 items), including self-reported health, perceived freedom, crime safety, security perceptions, and deprivation measures for food, medicine, and shelter; 4) Personal Values & Life Priorities (5 items), including the importance of family, friends, leisure, work, and social trust; 5) Religious & Spiritual Beliefs (5 items), including religion importance, God importance, religious service attendance, religious identity, and religion type; and 6) Political Trust & Civic Engagement (10 items), including politics importance, confidence in press, police, government, politics, universities, and elections, as well as corruption perceptions, democracy importance, and national pride (see *SI Appendix*, Table S1a for the complete list of covariates). These variables were then embedded directly into natural language profiles that served as the prompts to the models. Each profile explicitly described the respondent’s characteristics—for example:

“Based on the following information: Gender: Female, Age: 25 to 34, Education: Completed secondary school, Employment status: Full-time employed, Health: Good, Perceived freedom: 7/10, …, Country: Brazil. Overall, how satisfied is this person with their life nowadays? Please answer on a 0 to 10 scale, where zero means not satisfied at all and 10 means completely satisfied.”

These prompts were submitted to four state-of-the-art LLMs—GPT-4o mini, Claude 3.5 Haiku, LLaMA 3.3 70B, and Gemma 3 27B—via their respective APIs. Models were instructed to return a single numeric prediction on the 0 to 10 scale in JSON format. Predictions were generated deterministically (temperature = 0). Importantly, LLMs were never shown actual life satisfaction responses; their predictions relied only on the sociodemographic and attitudinal profiles together with the semantic and cultural associations embedded in pretraining corpora, not on statistical fitting to the WVS. To ensure equal cross-national representation, we randomly selected 1,000 respondents per country, yielding a total of N = 64,000 across 64 countries.

### Benchmarking against Statistical Models.

To benchmark the performance of LLMs, we conducted a standard out-of-sample prediction exercise. The full dataset was randomly split into an 80% training set and a 20% test set. On the training set, we estimated life satisfaction using two standard approaches:

• OLS: a linear regression of life satisfaction on the complete set of covariates listed in *SI Appendix*, Table S1a;
• Lasso Regression: a penalized linear model that automatically selects predictive features via L1 regularization.

These models were then used to generate predicted life satisfaction scores for individuals in the test set. Prediction accuracy was assessed using MAE, defined as the absolute difference between actual and predicted life satisfaction scores. Specifically, we computed MAE(OLS) and MAE(Lasso) for the statistical models. For each LLM, MAE(LLM) was calculated by comparing its predicted scores—derived directly from the natural-language profiles in the test set—against actual self-reports. Unlike OLS and Lasso, which estimate coefficients from the WVS sample, LLMs applied associations embedded in their pretraining corpora to generate predictions without access to life satisfaction outcomes. This comparison evaluates how well pretrained knowledge aligns with empirical survey relationships.

This component of the analysis—including the prediction pipeline, model comparisons, and evaluation metrics—was preregistered at https://aspredicted.org/3xsf-kx78.pdf.

### Structural Comparison of Predictors and Country Fixed Effects.

To assess whether LLMs replicate the structural relationships observed in life satisfaction data, we estimated five OLS regressions using the full sample of 64,000 individuals (1,000 per country): one with actual life satisfaction as the dependent variable and four with LLM-predicted life satisfaction from GPT, Claude, LLaMA, and Gemma. Each model included the same set of socioeconomic and attitudinal covariates listed in *SI Appendix*, Table S1a, allowing direct comparison of coefficients across models. Robust SE were used in all regressions.

All models also included country fixed effects to capture average differences in life satisfaction net of individual characteristics. The United States served as the reference category; because each country contributed equally to the dataset, this baseline choice is arbitrary and does not affect substantive interpretation. Robustness checks with alternative baselines (e.g., Germany, Mexico) yielded identical patterns. Fixed effects from each model were compared with observed data to evaluate how well LLMs reproduce cross-national variation. Coefficients from these analyses are visualized in Figs. 3 and 4 using STATA’s *coefplot*.

To examine how national context shapes systematic misprediction, we proceeded in two steps. First, for each LLM, we regressed the absolute prediction error on the same covariates used in the main OLS models, then averaged the fitted values within each country to yield a country-level measure of “explained error.” Second, we regressed this measure on four macroeconomic indicators—the Human Development Index (HDI), log GDP per capita, internet-user share, and the Gini coefficient—using publicly available data for 2017–2022. These indicators capture development (HDI, GDP), inequality (Gini), and digital exposure (internet penetration), the latter reflecting the likelihood of representation in LLM training data. Results are shown in Fig. 5*A* as scatter plots with fitted trend lines.

Finally, we plotted the country fixed effects themselves—which represent the unexplained, country-specific residuals (e.g., cultural, linguistic, institutional factors)—against the same macrolevel indicators. These associations are shown in Fig. 5*B*.

### Testing LLM Responses to Novel Policy-Like Inputs.

To examine whether LLMs rely on semantic similarity rather than reasoning from first principles, we designed a four-arm randomized experiment using synthetic variables and fabricated research papers. Each scenario involved a pair of fictitious variables with no true relationship to life satisfaction. One was arbitrarily framed as positive and the other as negative, with fabricated papers presenting empirical “evidence” of ±0.3 SD associations. Papers were written in the style of peer-reviewed publications (generated with OpenAI o3) and injected as context prompts.

Prompts were based on individual profiles from the WVS but included an added clause referencing one of the synthetic variables (e.g., *“…and has a dinosaur companion…”*). Variables were arranged along a semantic continuum using cosine similarity of embeddings (OpenAI text-embedding-3-large), ranging from the positively framed endpoint (condition 1) to the negatively framed endpoint (condition 7). Only the two endpoints were represented in the injected context; intermediate conditions (2–6) were unseen, providing a test of whether models interpolate smoothly along semantic gradients or remain flat under the null.

The design produced 4 scenarios × 7 conditions × 300 profiles per condition = 8,400 prompts per model. Models were randomized to one of four groups: i) Control (no fabricated research); ii) Placebo (injection with irrelevant synthetic variables); iii) Original Injection (fabricated papers linking condition 1 to higher and condition 7 to lower life satisfaction); and iv) Reverse Injection (same materials, but with directions inverted). Full stimulus materials and variable lists are provided in (*SI Appendix*, Fig. S8a shows cosine similarity matrices).

We compared predicted life satisfaction across models using the same set of prompts and used OLS to estimate treatment–control differences at each condition level via the following regression equation:

$$\begin{array}{c} {\mathrm{LS}}_{i}=\alpha +\beta_{1}{\mathrm{Treatment}}_{i}+\beta_{2}{\mathrm{Condition}}_{i}+\beta_{3}({\mathrm{Treatment}}_{i}\times {\mathrm{Condition}}_{i})\\ +\gamma +\varepsilon_{i}, \end{array}$$

where ${\mathrm{LS}}_{i}$ is LLM-predicted life satisfaction for individual *i*, ${\mathrm{Treatment}}_{i}$ is a binary indicator for assignment to the treatment group, and ${\mathrm{Condition}}_{i}$ is a categorical variable representing the semantic condition level. The model includes intervention fixed effects, *γ*, and $\varepsilon_{i}$ is the error term. The coefficient $\beta_{3}$ captures the treatment–control difference at each condition level. SE are clustered at the individual level to account for repeated observations.

Under the null hypothesis, significant differences should appear only at the injected endpoints (conditions 1 and 7), indicating that predictions shift only where trained. Systematic effects at the unseen intermediate conditions would instead suggest semantic generalization—extrapolation based on surface similarity of the injected clause. The reversed injection arm tests whether this extrapolation is directionally flexible, while the placebo condition disentangles content-specific effects from those due to generic exposure to academic-sounding text. Together, the four arms isolate the causal impact of injection on semantic generalization in LLMs (see *SI Appendix*, Fig. S4*A* for an illustration of the null hypothesis).

### Targeted Injection Experiment.

To test whether targeted context injection can mitigate systematic biases in LLM predictions, particularly in underrepresented countries, we ran a two-arm randomized controlled experiment. We focused on Claude, which consistently overestimated life satisfaction in sub-Saharan Africa. The treatment arm received the statement “Sub-Saharan African countries tend to report lower average life satisfaction compared to many other regions of the world” as contextual input. The control arm received no such injection. We then assessed whether this intervention reduced overestimation in sub-Saharan countries while leaving predictions for other regions unaffected.

We evaluated the method across three country groups: i) five accurately represented countries (Canada, Germany, Japan, Myanmar, and the United States), ii) four sub-Saharan countries (Ethiopia, Zimbabwe, Nigeria, and Kenya), and iii) five Latin American countries (Argentina, Brazil, Chile, Colombia, and Mexico). For each of the 14 countries, we generated 1,000 unique profiles (N = 15,000). Predictions were compared to actual self-reported life satisfaction and to baseline model outputs without injection.

Analytically, we re-estimated the full life satisfaction specification and extracted country fixed effects separately by treatment group. We also regressed absolute prediction error on covariates to obtain linear predictions and compared them across treatment arms. This approach allowed us to assess both whether injection improved calibration in targeted countries and whether it introduced unintended spillover effects in others.

### AI Usage.

AI tools, including ChatGPT (OpenAI) and Grammarly, were used to polish language, enhance clarity, and organize content in the manuscript. No AI tools were used to produce scientific findings or perform analysis. All outputs were carefully reviewed and verified by the authors.

## Supplementary Material

Appendix 01 (PDF)

## Data Availability

All LLM-predicted data and analysis code generated in this study are available at https://github.com/mitmedialab/wellbeing-LLM (35).
