# Supplementary material for: Simulating human well-being with large language models: Systematic validation and misestimation across 64,000 individuals from 64 countries
Source: Proc Natl Acad Sci U S A. 2025 Nov 26;122(48):e2519394122. doi: 10.1073/pnas.2519394122 (PMC12685116; doi:10.1073/pnas.2519394122)
Supplement: Supplementary file 1 — Appendix 01 (PDF) [file pnas.2519394122.sapp.pdf]

SI Appendix

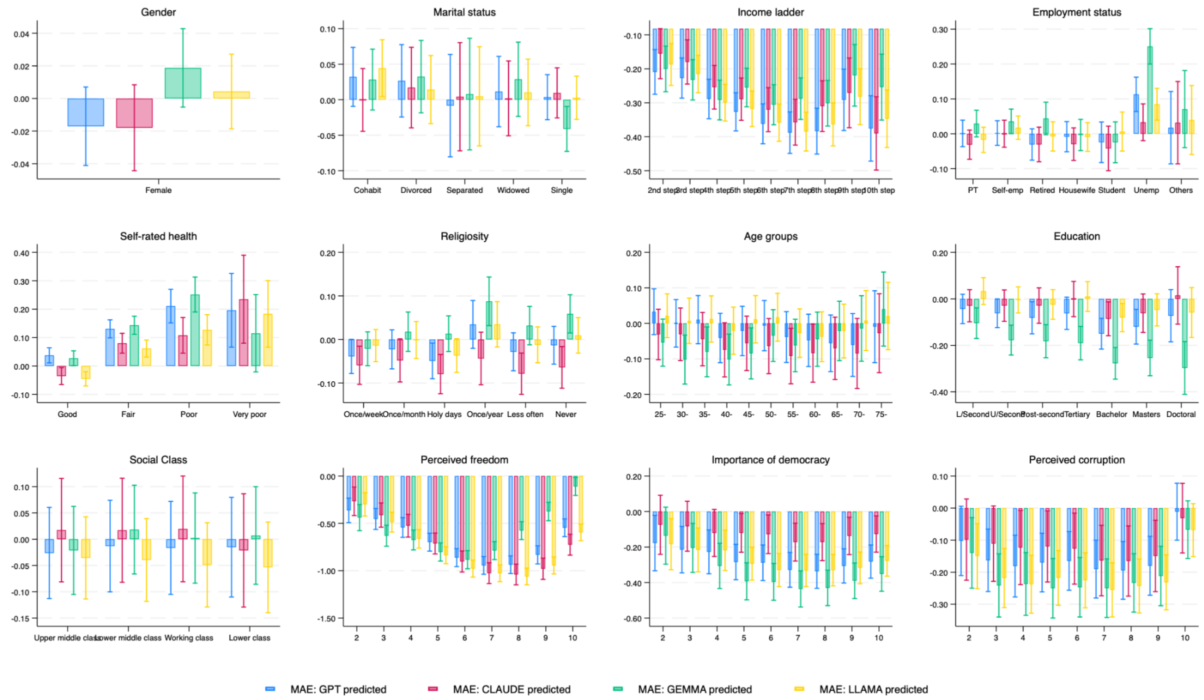

Fig 1a: Coefficient plot of covariates predicting the absolute difference between actual and LLM-predicted life satisfaction

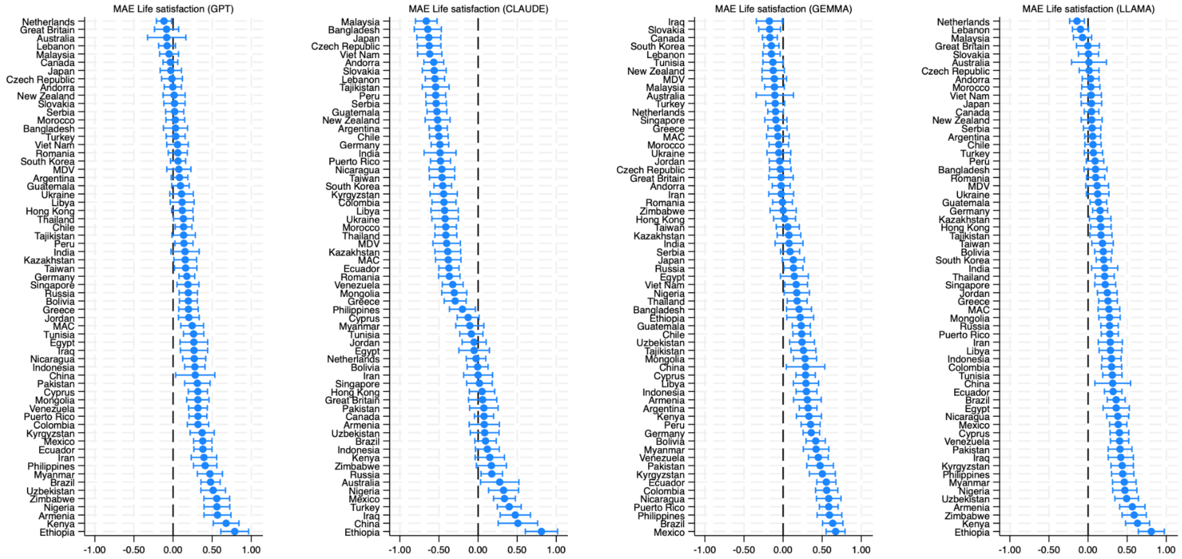

Fig 2a: Coefficient plot of country fixed effects from the absolute difference between actual and LLM-predicted life satisfaction.

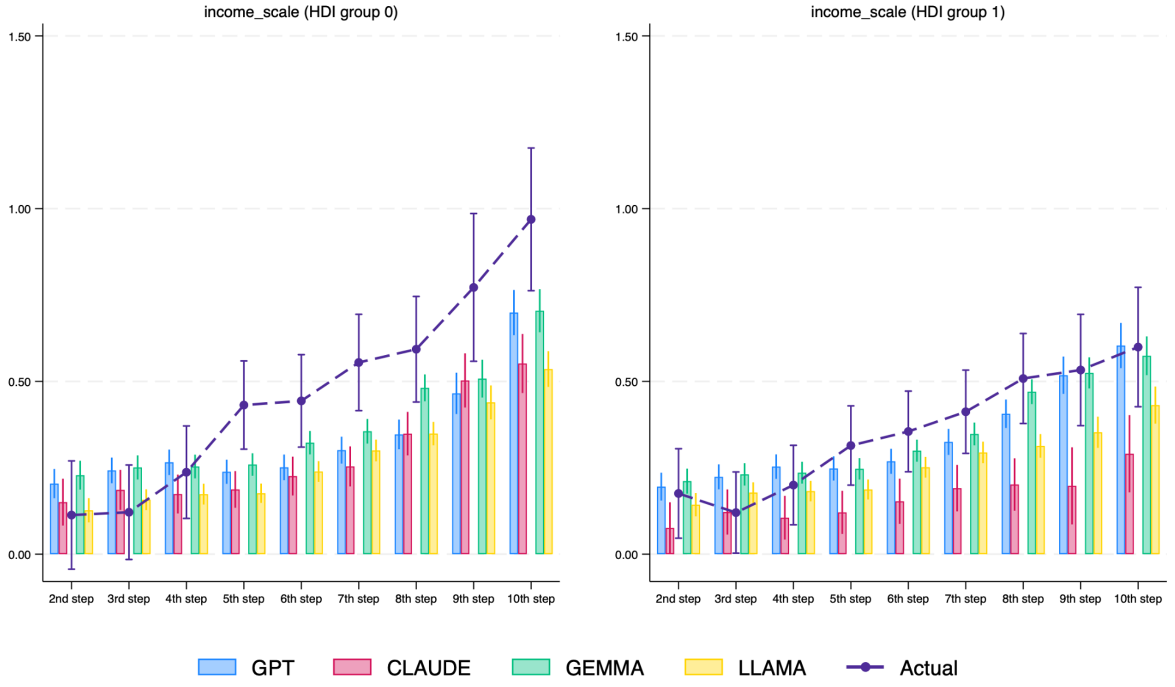

Figure 3a: The estimated relationship between income decile and life satisfaction across countries with below-median (left) and above-median (right) Human Development Index (HDI). The dotted lines represent the observed (actual) income–life satisfaction relationships with 95% confidence intervals, while the bars show predicted values from four large language models (GPT, Claude, Gemma, and LLaMA). Although the actual gradient is steeper in lower-HDI countries, all models estimate similar slopes across both groups—failing to capture the stronger role of income in less developed contexts.

Descriptive statistics

**Table 1a: Summary Statistics**					
**Dependent variables**	**N**	**Mean**	**SD**	**Min**	**Max**
Life satisfaction (actual)	63647	7.008	2.281	1	10
Life satisfaction (GPT)	64000	6.932	1.273	1	10
Life satisfaction (Claude)	64000	7.338	1.356	1	10
Life satisfaction (Gemma)	64998	6.004	1.192	2	10
Life satisfaction (Llama)	64998	6.953	1.071	1	10
**Covariates**	**N**	**Mean**	**SD**	**Min**	**Max**
Importance: Family (Q1)	63894	1.104	0.349	1	4
Importance: Friends (Q2)	63779	1.712	0.746	1	4
Importance: Leisure time (Q3)	63641	1.796	0.793	1	4
Importance: Politics (Q4)	63266	2.629	0.989	1	4
Importance: Work (Q5)	63248	1.529	0.770	1	4
Importance: Religion (Q6)	63378	1.907	1.059	1	4
Gender: Female (Q260)	63946	1.526	0.499	1	2
Age categories: 5-year intervals	63998	5.236	3.420	0	16
Self-reported health (Q47)	63827	2.189	0.880	1	5
Perceived freedom (Q48)	63409	7.187	2.291	1	10
Gone without food(Q51)	63654	3.484	0.856	1	4
Felt unsafe from crime (Q52)	63588	3.443	0.879	1	4
Gone without medicine (Q53)	63577	3.348	0.932	1	4
Gone without a cash income (Q54)	63587	3.156	1.014	1	4
Gone without a safe shelter (Q55	63470	3.718	0.702	1	4
Standard of living (Q56)	62825	1.737	0.864	1	3
Most people can be trusted (Q57)	63070	1.781	0.413	1	2
Confidence: The Press (Q66)	62446	2.714	0.876	1	4
Confidence: The Police (Q69)	62158	2.375	0.947	1	4
Confidence: The Government (Q71)	61628	2.664	0.987	1	4
Confidence: Politics (Q72)	61638	3.006	0.893	1	4
Confidence: Universities (Q75)	61305	2.170	0.858	1	4
Confidence: Election (Q76)	61174	2.642	0.958	1	4
Pref. income inequality (Q106)	63094	6.349	3.008	1	10
Perceptions of corruption (Q112)	62913	7.702	2.432	1	10
Perceptions of security (Q131)	63562	2.004	0.825	1	4
How important is God? (Q164)	62285	7.621	3.082	1	10
Religious service (Q171)	63253	4.072	2.179	1	7

Are you religious? (Q173)	62174	1.443	0.637	1	3
How important is democracy? (Q25	62856	8.352	2.149	1	10
Nation pride (Q254)	63133	1.593	0.863	1	5
Immigrant status (Q263)	63789	1.058	0.234	1	2
Citizen (Q269)	61778	1.024	0.152	1	2
Household size (Q270)	63362	4.009	2.281	1	63
Marital status (Q273)	63608	2.678	2.157	1	6
Number of children (Q274)	61775	1.799	1.743	0	22
Highest education (Q275)	63366	3.535	2.015	0	8
Employment status (Q279)	63264	3.141	2.063	1	8
Occupation (Q281)	59849	4.011	3.033	0	11
Working in the public sector? (Q284)	47920	1.821	0.562	1	3
Chief wage earner (Q285)	62107	1.538	0.499	1	2
Social class (Q287)	61378	3.238	0.975	1	5
Income scale (Q288)	62171	4.913	2.071	1	10
Religion (Q289)	63121	2.993	2.542	0	9
Literacy	30904	1.091	0.287	1	2
Settlement: Urban/rural	63855	3.059	1.509	1	5

Note: We included missing dummies for all variables in the regressions.

1. Personal Values & Life Priorities

1. Family importance (Q1)
2. Friends importance (Q2)
3. Leisure importance (Q3)
4. Work importance (Q5)
5. Social trust (Q57)

2. Religious & Spiritual Beliefs

1. Religion importance (Q6)
2. God importance (Q164)
3. Religious service (Q171)
4. Religious identity (Q173)
5. Religion type (Q289)

3. Political Trust & Civic Engagement

1. Politics importance (Q4)
2. Press confidence (Q66)
3. Police confidence (Q69)
4. Government confidence (Q71)
5. Politics confidence (Q72)
6. Universities confidence (Q75)
7. Election confidence (Q76)
8. Corruption perceptions (Q112)
9. Democracy importance (Q252)
10. National pride (Q254)

4. Economic Security & Social Status

1. Income deprivation (Q54)
2. Living standard (Q56)
3. Income inequality preference (Q106)
4. Employment status (Q279)
5. Occupation (Q281)
6. Public sector work (Q284)
7. Chief wage earner (Q285)
8. Social class (Q287)
9. Income scale (Q288)

5. Health & Personal Well-being

1. Self-reported health (Q47)
2. Perceived freedom (Q48)
3. Crime safety (Q52)
4. Security perceptions (Q131)
5. Food deprivation (Q51)
6. Medicine deprivation (Q53)
7. Shelter deprivation (Q55)

6. Demographic & Social Background

1. Gender (Q260)
2. Age categories
3. Immigrant status (Q263)

4. Citizenship (Q269)
5. Household size (Q270)
6. Marital status (Q273)
7. Number of children (Q274)
8. Education level (Q275)
9. Literacy
10. Urban/rural (Settlement)

Stimulus Materials for Semantic Generalisation Experiment

The four experimental scenarios, each consisting of a pair of variables and 5 in-between statements, are:

1. Scenario 1
    a. (Variable 1) Use origami birds as messengers
    b. Send paper animals as living mail
    c. Arrange talking paper mobiles
    d. Interact with moving paper sculptures
    e. Listen to decorative ceramic that moves
    f. Speak to house plants
    g. (Variable 2) Speak with household objects
2. Scenario 2
    a. (Variable 1) Wear only clothes made of moondust
    b. Dress in clothing made from stardust particles
    c. Wear garments that shift with emotions
    d. Consume drinks that make you glitter
    e. Dine on fruits that pulse with energy
    f. Taste meals that swirl with new flavors
    g. (Variable 1) Often eat food that changes color
3. Scenario 3
    a. (Variable 1) Drive a laughing car
    b. Ride a bicycle that tell funny stories
    c. Ride equipment that makes melodies
    d. Own garden tools that hum tunes
    e. Tend flower beds that whistle music
    f. Raise a plant that sing rock music
    g. (Variable 2) Adopt a family of singing mushrooms
4. Scenario 4
    a. (Variable 1) Listen to unicorn voices daily
    b. Hear messages from mythical beings

c. Detect whispers from fairy friends
d. Sense the secrets of legendary beings
e. Befriend creatures that speak riddles
f. Keep an ancient dragon as a pet
g. (Variable 2) Have a dinosaur companion

Only the two endpoints (conditions 1 and 7) were explicitly represented during injection. The intermediate conditions (2–6) were never seen by the model and thus serve as untrained transitions. Under the null hypothesis, the model should be indifferent to these intermediate values, and predicted life satisfaction should remain statistically flat across them. See Figure 8a for the cosine similarity matrix for seven statements in each experimental scenario.

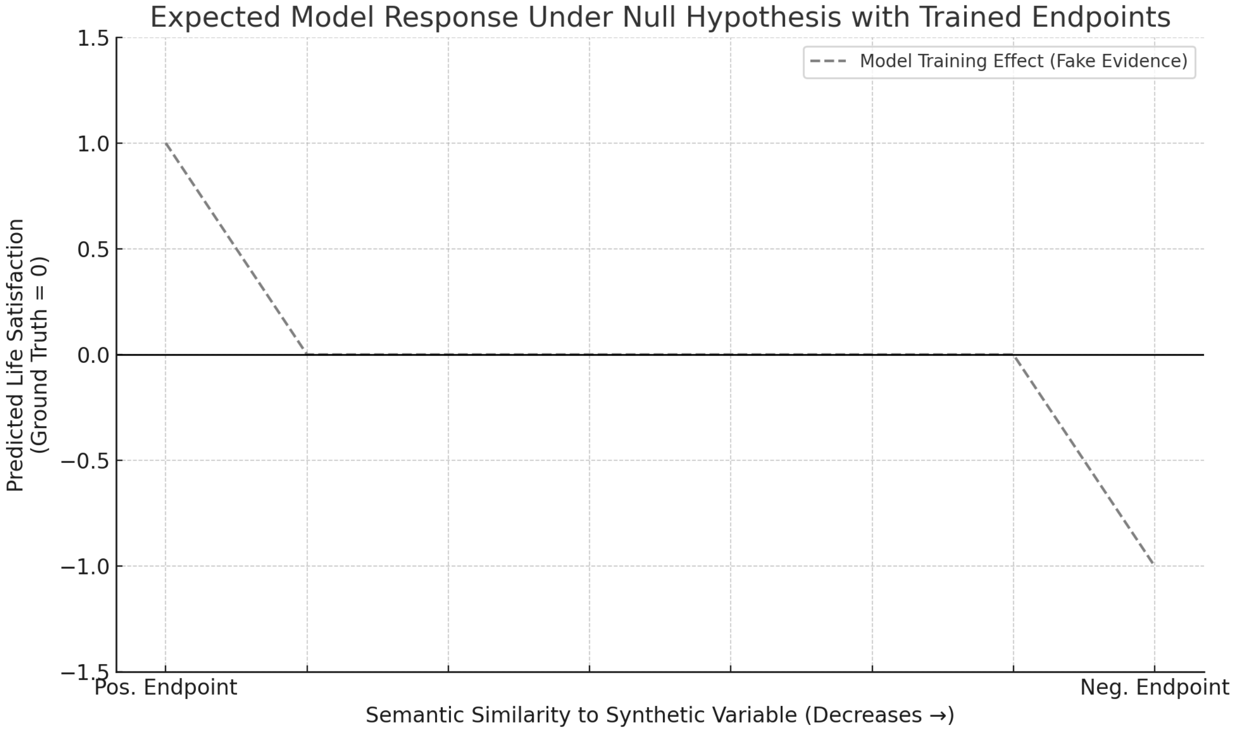

Fig 4a: Expected pattern of LLM-predicted life satisfaction across a semantic continuum. Under the null hypothesis, predicted life satisfaction should remain at zero for all transitional prompts. Due to context injection on synthetic papers, only the two endpoints were trained to have positive or negative associations with life satisfaction, producing deviations at the extremes despite a true underlying relationship of zero throughout.

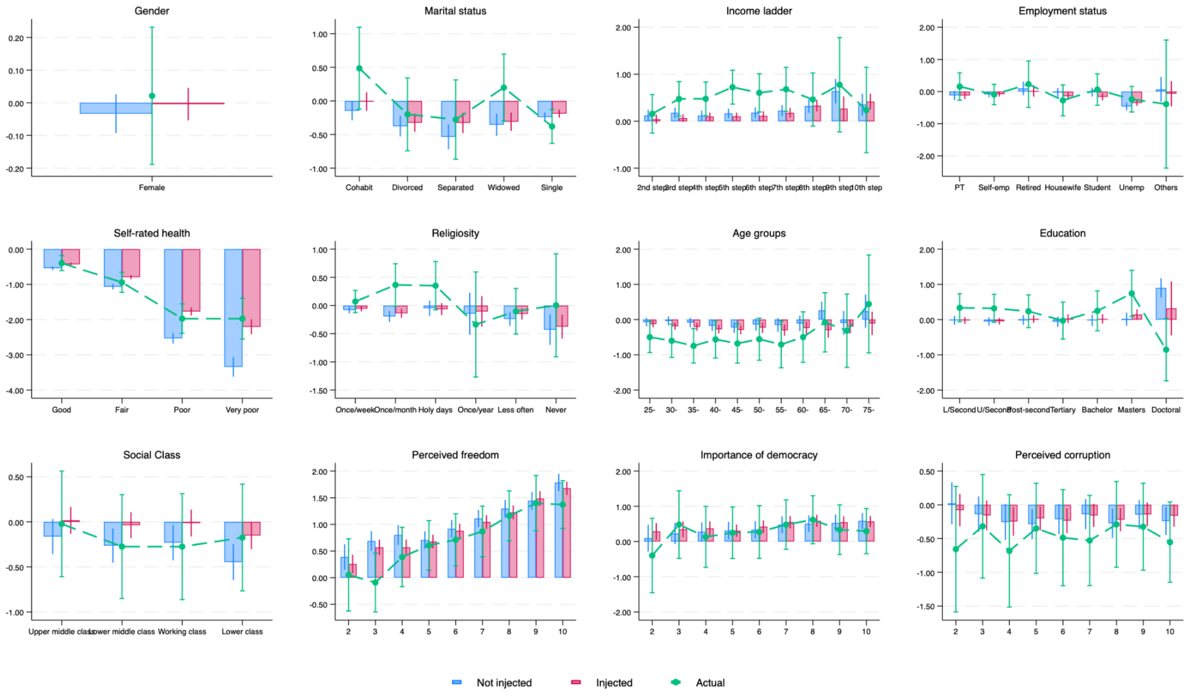

Fig 5a: Coefficient plot of socio-economic and attitudinal predictors of actual, non-injected, and injected LLM-predicted life satisfaction for Sub-Saharan countries: Ethiopia, Zimbabwe, Nigeria, and Kenya. Reference groups are: male (gender), married (marital status), first step (income ladder), full-time employed (employment status), very good (self-rated health), more than once a week (religiosity), younger than 25 (age groups), no formal education (education), upper class (social class), 1.little perceived freedom (perceived freedom), 1.democracy is not important (importance of democracy), and 1.zero corruption (perceived corruption). 95% confidence intervals are displayed.

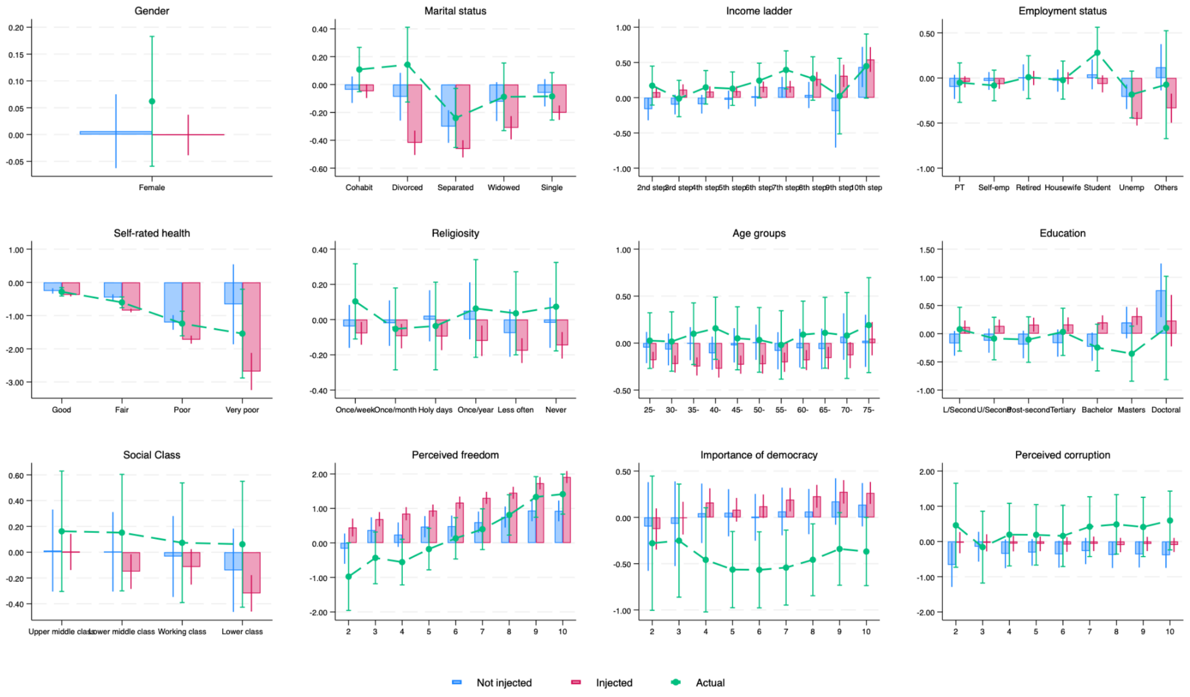

Fig 6a: Coefficient plot of socio-economic and attitudinal predictors of actual, non-injected, and injected LLM-predicted life satisfaction for Latin American countries: Argentina, Brazil, Chile, Colombia, and Mexico. Reference groups are: male (gender), married (marital status), first step (income ladder), full-time employed (employment status), very good (self-rated health), more than once a week (religiosity), younger than 25 (age groups), no formal education (education), upper class (social class), 1.little perceived freedom (perceived freedom), 1.democracy is not important (importance of democracy), and 1.zero corruption (perceived corruption). 95% confidence intervals are displayed.

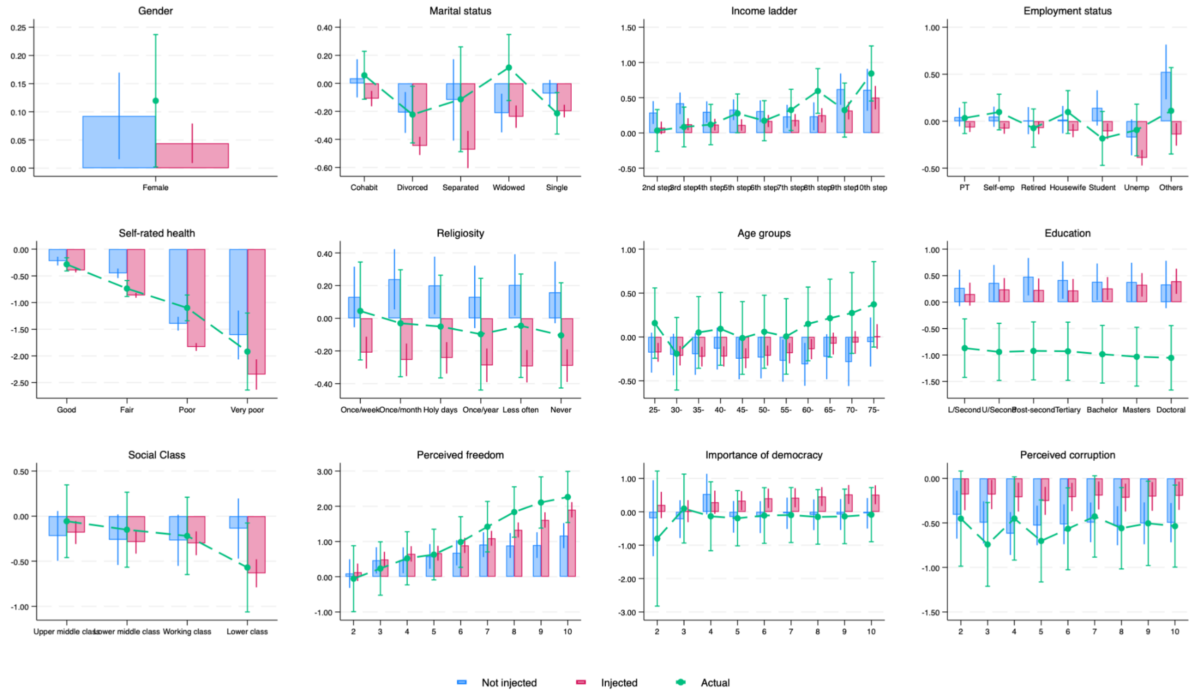

Fig 7a: Coefficient plot of socio-economic and attitudinal predictors of actual, non-injected, and injected LLM-predicted life satisfaction for 'Others' countries: Canada, Germany, Japan, Myanmar, and the United States. Reference groups are: male (gender), married (marital status), first step (income ladder), full-time employed (employment status), very good (self-rated health), more than once a week (religiosity), younger than 25 (age groups), no formal education (education), upper class (social class), 1.little perceived freedom (perceived freedom), 1.democracy is not important (importance of democracy), and 1.zero corruption (perceived corruption). 95% confidence intervals are displayed.

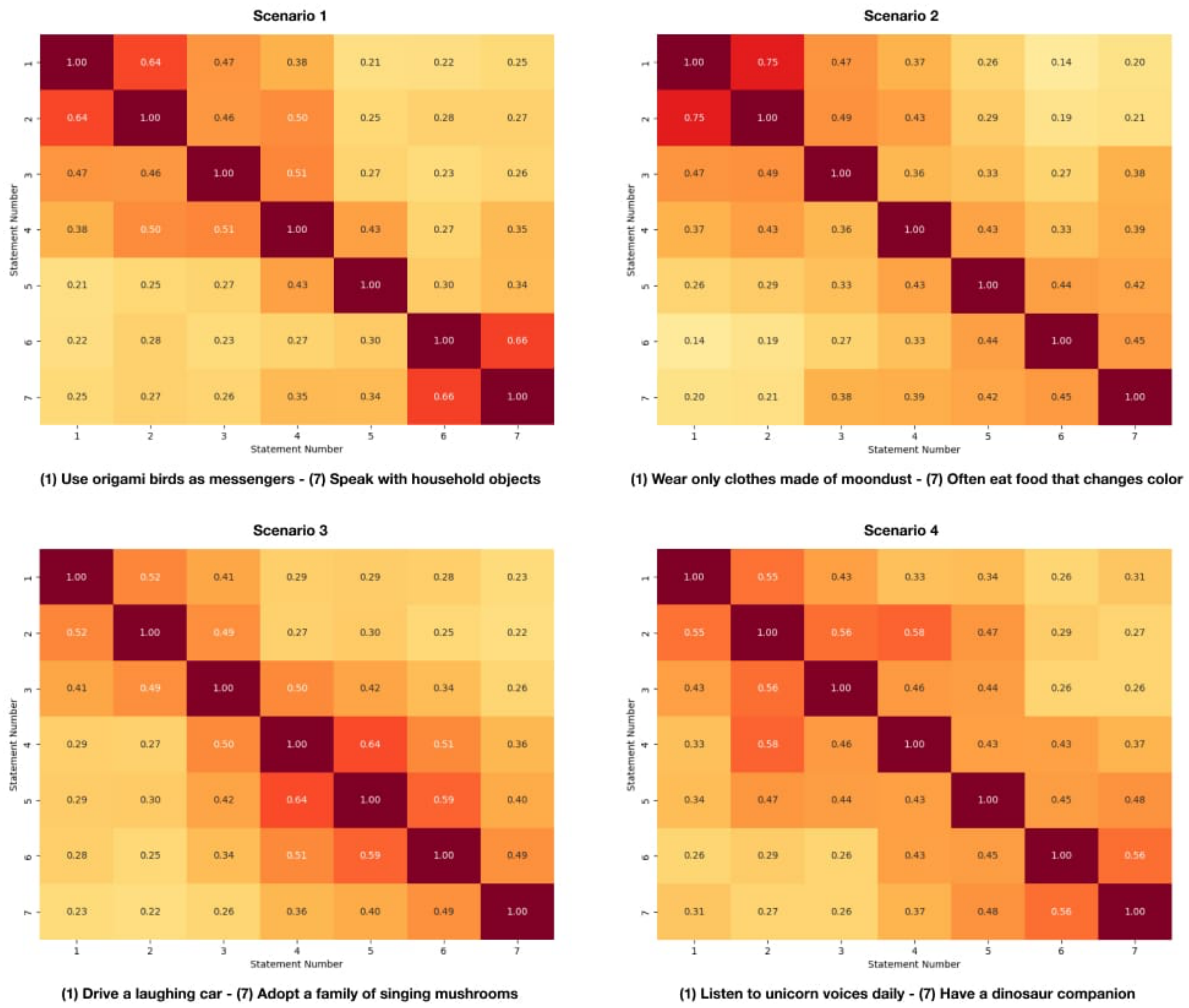

Figure 8a: Cosine similarity matrix for seven statements in each experimental scenario based on OpenAI text-embedding-3-large. The statements are designed to form a semantic continuum between the two endpoints (statement 1 and statement 7).
